# Risk factors contributing to tick-acaricide control failure in communal areas of the Oliver Tambo district eastern cape province, South Africa

**DOI:** 10.1007/s10493-024-00910-x

**Published:** 2024-04-24

**Authors:** William Diymba Dzemo, Oriel Thekisoe, Patrick Vudriko

**Affiliations:** 1 Department of Biological and Environmental Sciences, Faculty of Natural Sciences, Walter Sisulu University, NMD Private Bag X1, 5117 Mthatha, South Africa; 2 Unit for Environmental Sciences and Management, North West University, Potchefstroom, South Africa; 3 Research Center for Tropical Diseases and Vector Control, Department of Veterinary Pharmacy, Clinics and Comparative Medicine, College of Veterinary Medicine, Animal Resources and Biosecurity, Makerere University, Kampala, Uganda

**Keywords:** Cattle production, Tick control, Acaricide, Application practices, Control failure

## Abstract

Application of chemical acaricides in the control of ticks has led to the problem of tick-acaricide control failure. To obtain an understanding of the possible risk factors involved in this tick-acaricide control failure, this study investigated tick control practices on communal farms in the north-eastern part of the Eastern Cape Province (ECP) of South Africa. A semi-structured questionnaire designed to document specific farm attributes and acaricide usage practices was administered at 94 communal farms from the Oliver Tambo District municipality of the ECP. Data collected indicated that the main acaricide chemicals used at plunge dips of inland and coastal areas were synthetic pyrethroid formulations. Most (75%) farmers claimed not to have noticed a significant reduction in numbers of actively feeding and growing ticks on cattle after several acaricide treatments. Based on the farmers’ perceptions, leading factors that could have led to tick-acaricide control failure included: weak strength of the dip solution (76%); poor structural state of dip tanks (42%); and irregular tick control (21%). The rearing of crossbreeds of local and exotic cattle breeds, perceived weak strength of the dip solution and high frequency of acaricide treatment, were statistically associated with proportions of farms reporting tick-acaricide control failure. Furthermore, approximately 50% of farms reported at least four tick control malpractices, which could have resulted in the emergence and spread of tick-acaricide control failure. Other sub-optimal tick control practices encountered included incorrect acaricide rotation, and failure to treat all cattle in a herd. This data will inform and guide the development of management strategies for tick-acaricide control failure and resistance in communal farming areas.

**Supplementary Information:**

The online version contains supplementary material available at 10.1007/s10493-024-00910-x.

## Introduction

The communal farming sector provides the highest percentage (78%) of cattle within the Eastern Cape Province (ECP) of South Africa (Goni et al. 2018), yet the proportion of animals sold or consumed per year is low compared to the commercial farming sector (Musemwa et al. 2010). Ixodid ticks and tick-borne diseases (TBDs) have been highlighted as one of the major challenges facing communal cattle production (Moyo and Masika 2009; Sungirai et al. 2016; Goni et al. 2018,). Tick control on cattle in communal areas of the ECP is a free service provided for by the Provincial government through the District Veterinary Services (Moyo and Masika 2009). The Provincial government is responsible for the provision of acaricides, technical personnel and infrastructure. The control of ticks on cattle within the ECP is mainly through the use of plunge dips (Ntondini et al. 2008). However, tick-acaricide control failure has greatly moderated the efforts that have been made to control ticks and TBDs in South Africa (Moyo and Masika 2009). To lessen the effects of tick-acaricide control failure, farmers have often supplemented the government provided dipping services with their own initiatives including use of commercially accessible chemical acaricides applied as sprays and pour-ons, use of household disinfectants, engine oils, plant extracts, predation by chickens and manual removal (Moyo and Masika 2009).

Acaricide resistance which is a heritable reduction in susceptibility of a tick population to an acaricide expressed by repeated failure of the acaricide to achieve the expected level of control when used according to the label recommendation (FAO 2004; Rodriguez et al. 2018), is perhaps one of the several causes of acaricide control failure. The pace at which tick resistance develops is strongly influenced by poor acaricide application practices (Abbas et al. 2014). Acaricide resistance in field studies have been reported worldwide (Rodriguez et al. 2018). Several factors have been associated with increased probability of acaricide resistance and consequently acaricide control failure. These include frequency of application, type of application, farm localization, grazing management, cattle breed, incorrect dilution of acaricides, failure to treat all cattle in a herd and incorrect acaricide rotation (Spickett and Fivaz 1992; Moyo and Masika 2009; Vudriko et al. 2018; Rodriguez et al. 2018).

In the ECP of South Africa, studies have been conducted to assess the effectiveness of tick control practices in the commercial cattle production sector (Spickett and Fivaz 1992). However, in a study on tick control methods used by resource-limited farmers in communal cattle production areas, Moyo and Masika (2009) did not adequately investigate control practices associated with tick-acaricide control failure. Moreover, their study was conducted almost 10 years ago, and over time farming practices change, thus the need to assess the current tick control practices. Furthermore, in view of the reported wide spread prevalence of tick-acaricide control failure especially in communal areas of the Eastern Cape (Mekonnen et al. 2002; Moyo and Masika 2009), assessment of the current tick control practices is a prelude for the management of acaricide control failure in tick control programmes. Therefore, this study attempted to document farm attributes and control practices associated with the development of tick-acaricide control failure in communal areas of the ECP of South Africa.

## Materials and methods

### Study area and design

**Fig. 1:**
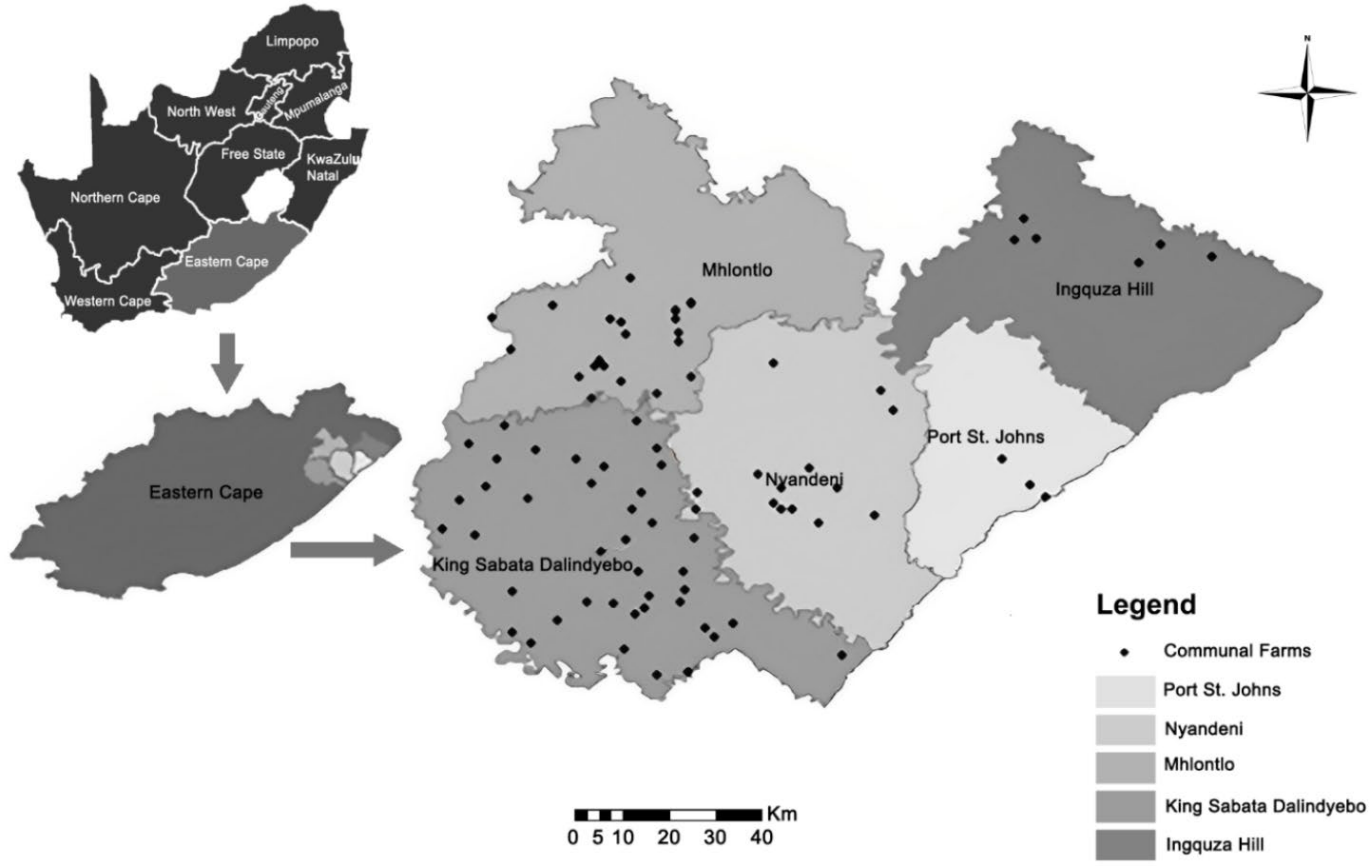
Map of OR Tambo district municipality showing locations of communal farms included in the study

The study was conducted from August 2018 to May 2019 at the Oliver Tambo District municipality (ORTDM), situated in the north-eastern part of the Eastern Cape Province of South Africa. There are five local municipalities (LM) within the ORTDM namely; King Sabata Dalindyebo (KSD), Mhlontlo, Nyandeni, Port St Johns and Ingquza (Fig. 1). All of these LMs, with the exception of KSD, are rural in nature with a subsistence economy. The ORTDM has the largest communal livestock farming in South Africa, incorporating 631 674 cattle, 732 478 goats and 1 225 244 sheep (IDP 2017). Communal areas comprise of villages, with land apportioned for residential, cropping and grazing purposes. Grazing areas are collectively shared by all villagers for rearing of different livestock including cattle, sheep, goats, horses and donkeys. Other agricultural activities practiced within the district include maize, vegetable, fruit and tea production, forestry, game rearing, apiculture and aquaculture. Most parts of the ORTDM receive an annual rainfall of above 900 mm and its environment has a wide range of habitats including inland and coastal grassland, Afromontane and coastal forest, valley thicket, thorny bushveld, coastal and marine habitats (IDP 2017).

There are approximately 355 dip tank stations (farms) within the ORTDM, of which 145 and 210 are located within the coastal and inland areas, respectively. Data on the number of dipping tanks and corresponding cattle population serviced at the various dip tanks were obtained from the Office of District Director in charge of Veterinary Services. Through a purposive sampling process based on the cattle population size (≥ 1000) serviced at the dipping tanks, a total of 148 inland and 72 coastal dipping tanks were selected, from which a random sample comprising 50% of the eligible dipping tanks was obtained. Hence for data collection, a total of 74 and 36 dipping tanks from the inland and coastal areas of the ORTDM, respectively, were considered.

### The survey instrument

A semi-structured questionnaire was used to document data on the attributes and control practices that could have resulted in the development of tick-acaricide control failure at the communal farms. The survey tool was designed to capture data on tick control methods and practices; acaricides used; animal husbandry and acaricide application practices; farmers’ perceptions on the causes of tick-acaricide control failure; and tick-acaricide control failure mitigation strategies. Tick-acaricide control failure was assessed by asking farmers if they had noticed a significant reduction in numbers of active ticks (feeding and growing) on cattle after several acaricide treatments (dipping). The survey tool was administered in the local isiXhosa language, since a majority of the communal farmers had little or no formal educational background and were not familiar with some terminologies used in the questionnaire. Prior to the administration of questionnaires, an informed oral consent was obtained from the Animal Health Technicians (AHTs), Community Animal Health Workers (CAHWs) and cattle farmers. Farmers were also assured of the confidentiality of their responses. Questionnaires were then administered to farmers on days of cattle treatment against ticks at the different dip tanks. The respondents were either delegated members of the dip tank committee (also cattle owners) or a group of cattle farmers who had finished the task of dipping the cattle. With the latter, a common consensus was reached before responses were recorded.

### Data analysis

Data obtained from the questionnaires was summarised, coded and entered into Microsoft® Excel® 2016. Counts, frequencies and percentages were generated and analysed in GraphPad Prism 8 for Windows version 8.01. The Fischer’s Exact probability test was used to determine whether risky tick control practices were significantly (two tailed *P value* ≤ 0.05) associated with proportion of farms reporting tick-acaricide control failure. Furthermore, Venn diagrams were used to illustrate the proportion of farms with combinations of multiple risk factors associated with tick-acaricide control failure.

## Results

### General characteristics of the communal cattle farms

86% (94/110) of the sampled communal farms participated in the survey, of which a majority (43%) were from the KSD local municipality (Table 1). Respondents were either a group of ordinary cattle owners (49%) or members of the dipping tank committee (51%), who had no other formal jobs (96%) apart from livestock rearing. Cattle breeds mostly kept were crossbreeds of the local Nguni and exotic breeds (68%). Cattle were kept alongside other livestock including sheep (88%), goats (73%), and horses (54%). All these ruminants were either bred on grassland vegetation (63%), or both grassland and forest vegetation (45%). Cattle interaction with wildlife in the course of feeding was minimal (23%).

**Table 1:** General characteristics of the studied communal cattle farming areas within OR Tambo district of South Africa

Query	Level	Percentage reponse per area		Percent of total (*n* = 94)
		Inland (*n* = 65)^#^	Coastal (*n* = 29)	
Local municipality	KSD	40 (26)	45 (13)	41 (39)
	Mhlonto	35 (23)	0 (0)	25 (23)
	Port St Johns	0 (0)	10 (3)	3 (3)
	Ingquza hill	10 (6)	17 (5)	12 (11)
	Nyandeni	15 (10)	28 (8)	19 (18)
Position at the communal farm	Cattle farmer	41 (27)	66 (19)	49 (46)
	Dipping tank committee member	59 (38)	35 (10)	51 (48)
Breed of cattle reared	*Crossbreeds only	63 (41)	79 (23)	68 (64)
	Both Local and crossbreeds	37 (24)	21 (6)	32 (30)
^a^Keeping of other livestock	Sheep	89 (58)	86 (25)	88 (83)
	Goat	66 (43)	90 (26)	73 (69)
	Horses	45 (29)	76 (22)	54 (51)
	Pigs	20 (13)	24 (7)	21 (20)
^b^Vegetation cattle feed on	Grassland only	72 (47)	55 (16)	67 (63)
	Grassland and forest	37 (24)	62 (18)	44 (42)
Interaction between cattle and wildlife	Yes	14 (9)	45 (13)	23 (22)
	No	86 (56)	55 (16)	77 (72)

*Abbreviation* KSD, King Sabata Dalindyebo

* Crossbreeds of the local Nguni and exotic cattle

^#^Numbers in brackets indicate number of responses

^a^Some farmers had a combination of other livestock, and combinations of livestock were not accounted for

^b^Some farmers grazed their cattle in both grassland-only and grassland-and-forest areas

### Cattle ticks, their effects and control

A majority (95%) of farmers indicated that their cattle were infested with ticks. Based on the blue colour of engorged female ticks, as well as coloured patterns on scutum and coloured rings on legs of bont-legged ticks, *Rhipicephalus (Boophilus)* spp. (95%) and *Amblyomma* spp. (55%) were mostly reported (Table 2). Bont-legged ticks were reportedly present on majority cattle (83%) kept at the coastal communal areas. Furthermore, farmers indicated that greater number of active ticks were found on cattle during the summer (86%). With regards to the effects of ticks, a majority (95%) of farmers knew that tick infestations led to weight loss, TBDs, and wounds on body, ears, udder, teat and tail of the animal. In addition, some farmers mentioned that tick infestations also lead to abscesses (28%) and loss of appetite (5%) in cattle. Tick control at the communal farms was conducted mainly by immersing cattle into an acaricide solution in a dip tank. Other tick control measures encountered included: the use of home-made remedies (7%); predation by chickens (11%) and handpicking (2%). Furthermore, in a few instances, commercially available chemical products were reportedly used to complement the dipping of cattle, in the form of hand sprays, pour-ons and injections with ivermectin (Table 2). Chemical spraying was carried using a 5–10 L hand operated sprayer (2%) or locally fabricated 2 L hand operated sprayers (4%).

**Table 2:** Tick control on cattle in communal farming areas of the OR Tambo district of South Africa

Query	Level	Percentage response per area		Percent of total (*n* = 94)
		Inland (*n* = 65)^#^	Coastal (*n* = 29)	
Presence of cattle ticks	Yes	95 (62)	93 (27)	95 (89)
	No	5 (3)	7 (2)	5 (5)
*Common name of the ticks	Blue ticks Bont legged ticks	95 (62) 43 (28)	93 (27) 83 (24)	95 (89) 55 (52)
*Effects of ticks on cattle	Weight loss Wounds on body, ears, udder, teat and tail Tick borne diseases Abscesses	95 (62) 95 (62) 95 (62) 15 (10)	93 (27) 93 (27) 93 (27) 55 (16)	95 (89) 95 (89) 95 (89) 28 (26)
	Loss of appetite	5 (3)	7 (2)	5 (5)
Time of the year with high tick infestations	Summer	88 (57)	83 (24)	86 (81)
	Winter	5 (3)	7 (2)	5 (5)
	Whole year	3 (2)	3 (1)	3 (3)
	Note sure	5 (3)	7 (2)	5 (5)
*Tick control methods	Handpicking	2 (1)	3 (1)	2 (2)
	Predation by chickens	11 (7)	14 (4)	12 (11)
	Chemicals (acaricides)	100 (65)	100 (29)	100 (94)
	Home-made remedies	8 (5)	7 (2)	7 (7)
*Acaricide application method	Plunge dip	100 (65)	100 (29)	100 (94)
	Hand spraying	2 (1)	17 (5)	6 (6)
	Pour-ons	14 (9)	14 (4)	14 (13)
	Injectables	3 (2)	17 (5)	7 (7)
*Facility/equipment used for acaricide application	Dipping tank	100 (65)	100 (29)	100 (94)
	Hand operated sprayer (5–10 L)	0 (0)	7 (2)	2 (2)
	Syringe	3 (2)	17 (5)	7 (7)
	Lid-perforated 2 L plastic bottle	2 (1)	10 (3)	4 (4)

^#^Numbers in brackets indicate number of responses

* Several farmers offered multiple responses to the query statement

**Fig. 2:**
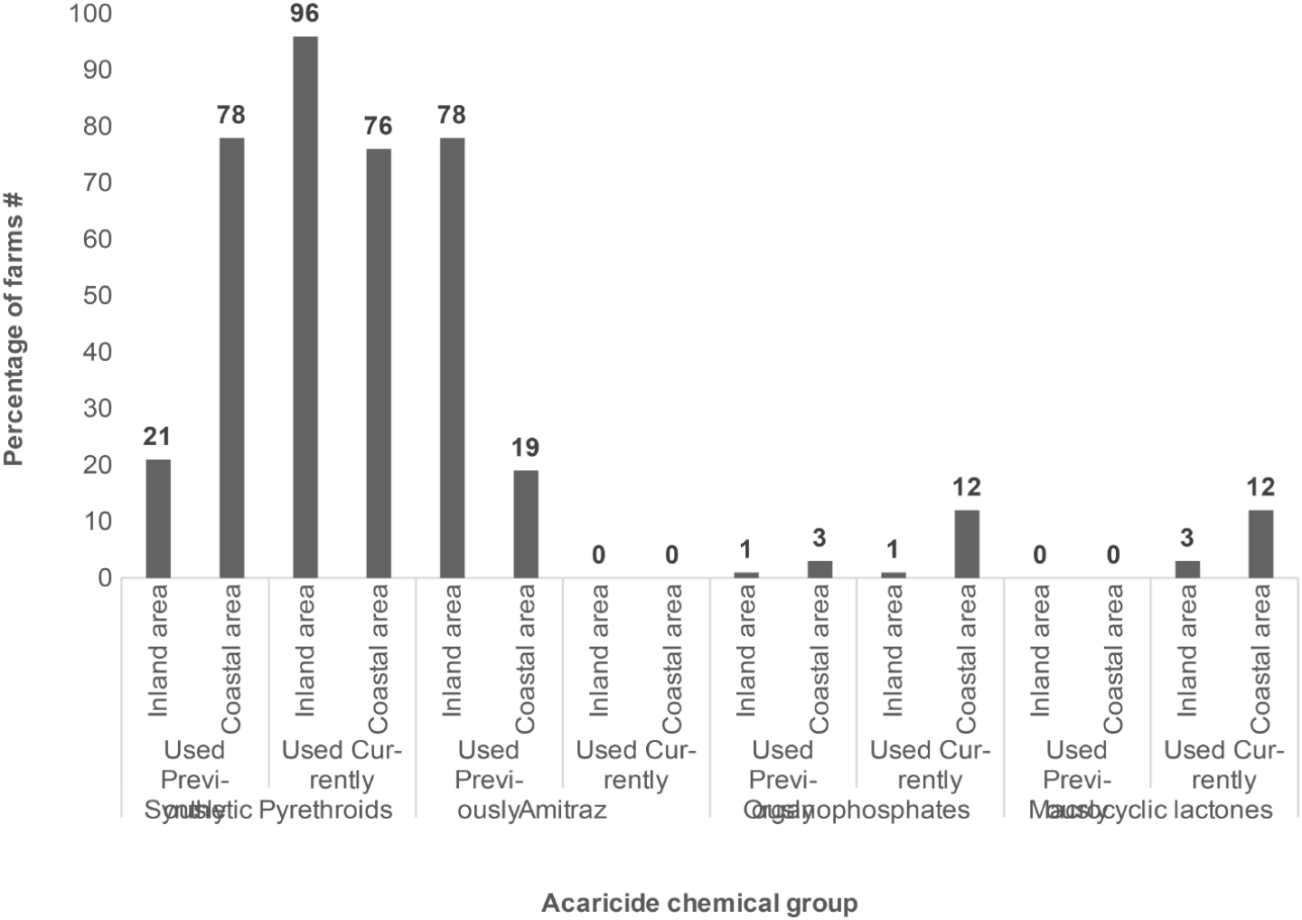
Acaricide chemical groups used on cattle ticks on communal areas in the OR Tambo District of the Eastern Cape province of South Africa. ^#^ Percentages were obtained from number of farms that used an acaricide chemical group and total number of farms sampled

A total of 10 acaricide brands were mentioned to have been used in the control cattle ticks (Table 3). Acaricide formulations, provided by the government that have been used previously in plunge dips at communal farms of the ORTDM included: Triatix® (Amitraz 12.5% m/v, a formamidine), Deca-tix®3 (Deltamethrin, 2.5% m/v, a synthetic pyrethroid) and, Delete-X5® (Deltamethrin, 5% m/v, a synthethic pyrethroid) (Fig. 2). A considerably small number of farms had previously used synthetic pyrethroids (Deadline®) and organophosphates (Supadip®) to complement the government funded dipping services. Deca-tix® and Delete-X5® were more recently being used at 71% and 21% of the inland-located dip tanks, respectively. However, all the coastally located dip tanks were using Delete-X5® (Table 3). To complement cattle dipping, farmers used macrocyclic lactone (ML) and organophosphate (OP) formulations (Fig. 2).

**Table 3:** Past and present use of acaricides in the control of ticks on cattle in communal areas

Query	Level	Percentage response per area		Percent of total (*n* = 94)
		Inland (*n* = 65)^#^	Coastal (*n* = 29)	
*Acaricides previously used	Deca-tix (Plunge dip)	12 (8)	76 (22)	32 (30)
	Delete -X5 (Plunge dip)	8 (5)	3 (1)	6 (6)
	Triatix (Plunge dip)	80 (52)	21 (6)	62 (58)
	Deadline (Pour on) Supadip (Spray)	2 (1) 2 (1)	7 (2) 3 (1)	3 (3) 2 (2)
Duration of acaricides used previously				
	1–2 years	2 (1)	0 (0)	1 (1)
	2–3 years	11 (7)	0 (0)	8(7)
	Above 3 years	17 (11)	72 (21)	34 (32)
	Note sure	70(46)	28 (8)	57 (54)
*Names of acaricides in use currently	Deca-tix (Plunge dip)	71 (46)	0 (0)	49 (46)
	Delete -X5 (Plunge dip)	29 (19)	100 (29)	51 (48)
	Deadline (Pour on)	9 (6)	7 (2)	9 (8)
	Maxipour (Pour on)	5 (3)	3 (2)	5 (5)
	Dectomax (Injectable)	2 (1)	7 (2)	3 (3)
	Ecomectin (Injectable)	0 (0)	3 (1)	1 (1)
	Virbamec (Injectable)	2 (1)	7 (2)	3 (2)
	Supona aerosol (Spray) Supadip (Spray)	0 (0) 2 (1)	7 (2) 10 (3)	2 (2) 4 (4)
Duration of acaricides used currently	Less than 1 year	2 (1)	0 (0)	1 (1)
	1–2 years	21 (14)	3 (1)	16 (15)
	2–3 years	2 (1)	14 (4)	5 (5)
	Above 3 years	63 (41)	66 (19)	64 (60)
	Note sure	12 (8)	17 (5)	14 (13)
*Reasons for changing to the currently used acaricides.	AHT advice	100 (65)	100(29)	100(94)
	Ticks not dying	74 (48)	76(22)	75 (70)
	Poor state of dipping tank Lack/shortage of water	8 (5) 22 (14)	10 (3) 35 (10)	9 (8) 26 (24)
Reduction in numbers of active ticks on cattle after dipping.	Sometimes	26 (17)	24 (7)	25 (24)
	No	74 (48)	76 (22)	75 (70)

*Abbreviation* AHT, Animal Health Technician

* Several farmers offered multiple responses to the query statement

^#^Numbers in brackets indicate number of responses

It was observed that most farmers (57%) could not remember the exact usage-duration of the previously applied acaricides. However, most of them (64%) reported to have been using the currently applied acaricides for a duration of above 3 years (Table 3). Advice on the rotation or change of acaricide at the farm was mainly provided by the AHT (100%). According to the farmers, the acaricides were changed or rotated because the ticks are not dying (75%). A majority (75%) of farmers reportedly did not observe a significant reduction in numbers of active ticks (feeding and growing) on cattle after several acaricide applications (tick-acaricide control failure).

### Farmers’ perceptions on the causes of tick-acaricide control failure

According to the farmers, tick-acaricide control failure could have been caused by a number of factors (Table 4). A majority (76%) of them indicated that the strength of the acaricide mixture inside the dip tank was weak and ineffective in killing ticks. Poor structural state of the dip tanks (42%) and irregular cattle tick control (21%) were also frequently mentioned. They noted that the alleged weak strength of the acaricide mixture in the dip tank might have been as a consequence of mud accumulation (21%) and high water level (14%) after heavy rains (Table 4). Other causes of tick-acaricide control failure mentioned included; limited quantity of the provided acaricide solution, and closeness to the forest. Most farmers (84%) were against the fact that tick-acaricide control failure coincided with the arrival of new animals. Whenever tick-acaricide control failure was observed most farmers (94%) would report and seek advice from the AHT. To reduce or minimize tick-acaricide control failure, farmers demanded for an increase in the strength of the acaricide mixture in the dip tank (85%) or replacement of the acaricide (20%).

**Table 4:** Farmers’ perceptions about the causes and management of tick-acaricide control failure on communal areas of the Eastern Cape Province

Query	Level	Percentage response per area		Percent of total (*n* = 94)
		Inland (*n* = 65)^#^	Coastal (*n* = 29)	
* Causes of tick-acaricide control failure	Poor state of dip tank	28 (18)	76 (21)	42 (39)
	Weak acaricide mixture	77 (50)	72 (21)	76 (71)
	Irregular cattle tick control	22 (14)	21 (6)	21 (20)
	Mud inside the dip tank	2 (1)	21 (6)	7 (7)
	Shortage of acaricide solution	5 (3)	7 (2)	5 (5)
	Water level inside dip tank after heavy rains	0 (0)	14 (4)	4 (4)
	Closeness to forest	2 (1)	3.4 (1)	2 (2)
Acaricide failure coincides with arrival of new animals	Yes	19(12)	10 (3)	16 (15)
	No	81 (53)	90 (26)	84 (79)
* Sources of advice whenever acaricide failure is observed	Vet shop teller	17 (11)	21 (6)	18 (17)
	CAHW/ fellow farmer	12 (8)	3 (1)	10 (9)
	AHT	95 (62)	90 (26)	94 (88)
* What should be done when acaricide failure is observed?	Increase the strength of acaricide mixture	91 (59)	72 (21)	85 (80)
	Use another acaricide	17 (11)	28 (8)	20(19)
Community discusses strategies to curb acaricide failure	Yes	46 (30)	35 (10)	43 (40)
	No	54 (35)	65 (19)	57 (54)

*Abbreviations* CAHW, Community Animal Health Worker; AHT, Animal Health Technician

* Several farmers offered multiple responses to the query statement

^#^Numbers in brackets indicate number of responses

### Tick-acaricide control application practices

Acaricide solutions provided for by the Eastern Cape Provincial government were supplied to the communal areas through the AHT (95%), CAHWs or dipping committee members (12%). At a few farms (18%), farmers complemented dipping with commercially available acaricide chemical products that were obtained from nearby veterinary drug shops or supermarkets (Table 5). The AHTs (98%) provided advice on all tick control related issues. However, a few farmers consulted the sales persons at drug shops or supermarkets, dipping committee members, or simply relied on their own personal experiences. Often (95%), the mixing of the acaricide solution was conducted by an appointed dipping committee member, most (72%) of whom had received basic training on acaricide application techniques. With regard to acaricide treatment frequency, most cattle (96%) were treated at the recommended rate of 2–4 times per month (24–48 times/year) during the summer. However, during the winter season, irregular acaricide application was rife (91%). It was also noted that most farmers (89%) did not always bring all their cattle for treatment against ticks. Moreover, it was noted that not all (43%) of the newly introduced cattle into the communal area were treated before integrating them with other animals. In communal grazing areas, interactions between cattle and other livestock species including goats, sheep, and horses is inevitable. Consequently, a high level (99%) of interaction between treated cattle and these livestock species was predictable (Table 5).

**Table 5:** Tick-acaricide control application practices on communal areas

Query	Level	Percentage response per area		Percent of total (*n* = 94)
		Inland (*n* = 65)^#^	Coastal (*n* = 29)	
*Sources of the acaricides	Vet. drug shop	14(9)	14(4)	14 (13)
	Supermarket	5(3)	3(1)	4(4)
	AHT	99(64)	86(25)	95(89)
	CAHWs/ Dipping committee member	11(7)	14(4)	12(11)
*Sources of advice on tick control	Vet. drug shop	11(7)	14(4)	12(11)
	AHT	100(65)	93(27)	98(92)
	Dipping committee member	1(1)	3(1)	2(2)
	Personal experience	1(1)	7(2)	3(3)
Who mixes the acaricides	Appointed dipping committee member	95(62)	93 (27)	95(89)
	Cattle owner	5(3)	7(2)	5(5)
Person who mixes the acaricides has been trained	Yes	66(43)	86(25)	72(68)
	No	34(22)	14 (4)	28 (26)
Acaricide application frequency during summer	2–4 times per month	95 (62)	97 (28)	96 (90)
	once a month	5 (3)	3 (1)	4 (4)
Acaricide application frequency during winter				
	Once a month	0 (0)	28 (8)	9 (8)
	Inconsistent	100 (65)	72 (21)	91 (86)
Farmers bring all the cattle, always for treatment	Yes	14 (9)	3 (1)	11 (10)
	No	86(56)	97 (28)	89 (84)
Interaction between treated cattle and other livestock species	Yes	97 (63)	100 (29)	99 (92)
	No	3(2)	0 (0)	1(2)
Newly introduced cattle into the communal area are treated	Yes	54 (35)	55 (16)	54 (51)
	No	45(29)	38 (11)	43 (40)
	Sometimes	1(1)	7 (2)	3 (3)

*Abbreviations* CAHW, Community Animal Health Worker; AHT, Animal Health Technician

* Several farmers offered multiple responses to the query statement

^#^Numbers in brackets indicate number of responses

### Farm attributes and practices associated with the development of tick-acaricide control failure

**Fig. 3:**
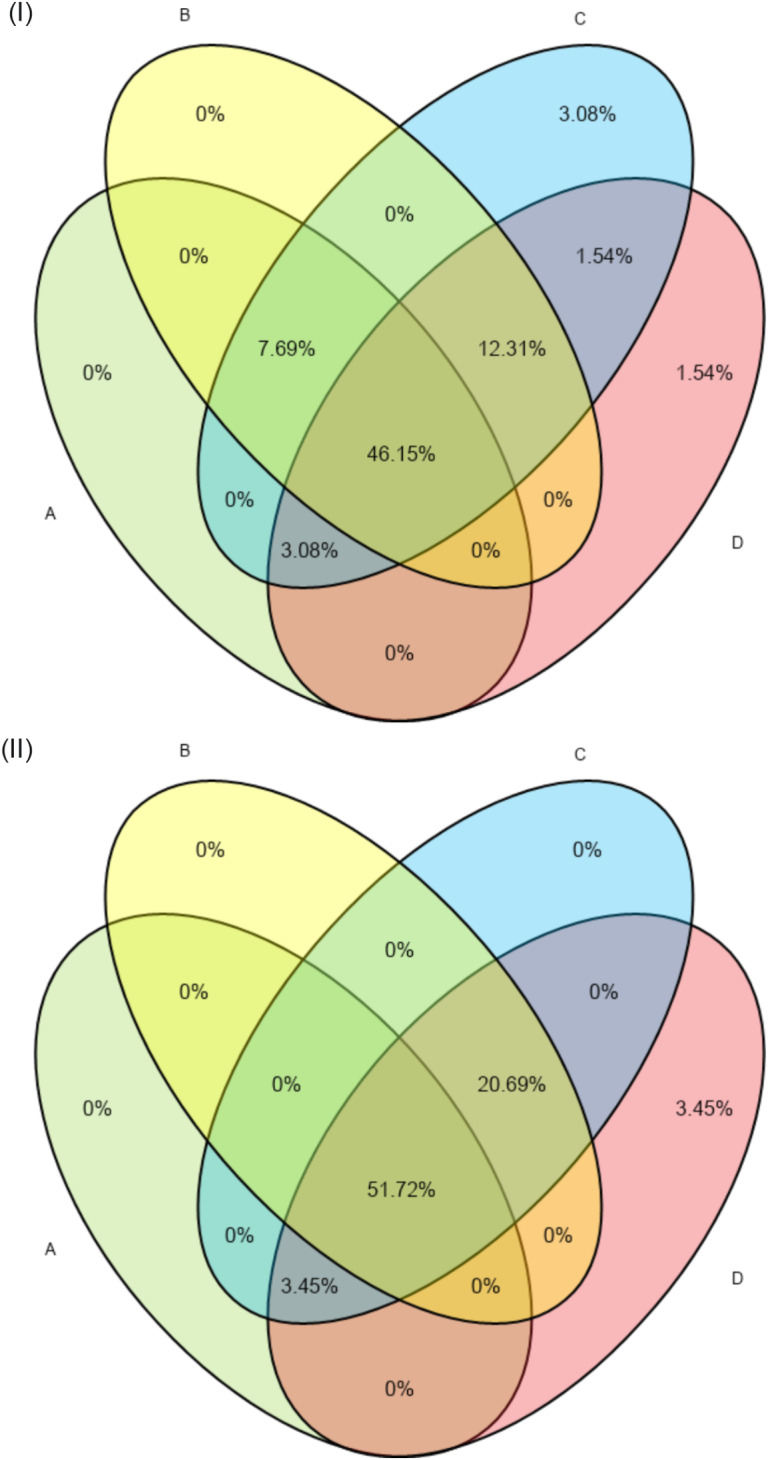
Proportions of Inland (I) and Coastal (II) communal farms with combinations of risk factors associated with tick-acaricide control failure. A- Alleged weak strength of the dip solution; B- Irregular tick control; C- High acaricide application frequency during summer; D- Failure to treat all cattle in a herd

There were statistically significant associations (*P* ≤ 0.05) between rearing of non-descript cattle breeds, alleged weak strength of acaricide mixture inside the dip tank, acaricide treatment frequency during summer, and proportion of farms reporting high tick-acaricide control failure (Table 6). There was insufficient research evidence to associate reported tick-acaricide control failure with geography, failure to treat all cattle in a herd, irregular tick control and poor structural state of the dip tanks (Table 6). An overall proportion of 46.15% and 51.72% of the inland and coastal farms, respectively, reported at least four risky acaricide application practices, which could have resulted into tick-acaricide control failure (Fig. 3). These included the alleged weak strength of the diluted acaricide mixture in the dip tank; irregular tick control; high frequency of acaricide treatment during summer; and failure to treat all cattle in a herd.

**Table 6:** Analysis of control malpractices associated with tick-acaricide control failure on communal areas

Factor	Response level	Tick-acaricide control failure reported on the farm		Fisher Exact probability test value
		Yes (*n* = 70)	No (*n* = 24)	
Geographical location	Inland Coastal	48 22	17 7	*P* = 1.00
Cattle breed	*Crossbreeds only	59	4	*P* < 0.0001
	Local & crossbreeds	11	20	
Frequency of acaricide treatment in summer	2–4 times/month	69	21	*P* = 0.049
	Once a month	1	3	
Alleged weak strength of the dip solution inside the dip tank	Yes	61	10	*P* < 0.0001
	No	9	14	
Failure to treat all cattle in a herd	Yes	69	23	*P* = 0.447
	No	1	1	
Irregular tick control	Yes	64	20	*P* = 0.271
	No	6	4	
Poor state of dip tank	Yes	32	7	*P* = 0.229
	No	38	17	

* Crossbreeds of the local Nguni and exotic cattle

## Discussion

Several farm management and tick control malpractices reportedly associated with increased probability of tick-acaricide control failure have been identified in this study. On communal and commercial (Spickett and Fivaz 1992) farming areas of the Eastern Cape Province of South Africa, rearing of non-descript cattle breeds (mainly Bonsmara) is preferred to the local Nguni cattle. As a result, the herd composition on communal farms is mainly made up of the highly productive and tick-susceptible crossbreeds of the local Nguni and exotic breeds. Therefore, we speculate that the rearing of these cattle crossbreeds is related to the reported tick-acaricide control failure, as they have been found to harbour significantly more ticks (species and numbers) than the local indigenous Nguni cattle breeds (Nyangiwe et al. 2011). Additionally, the Nguni cattle is known for its high adaptability to challenging local environmental conditions, and through natural selection it has acquired a higher degree of tick and disease resistance (Spickett et al. 1989; Norval 1994; Bester et al. 2001). Hence, the keeping of indigenous cattle breed is being promoted as an option for the integrated control of ticks and tick-borne diseases in South Africa (Spickett and Fivaz 1992).

The use of chemical products has been the main control intervention against cattle tick infestations worldwide (Abbas et al. 2014). Plunge dipping of cattle into an acaricide solution is the main control method against ticks and TBDs in communal areas of the ECP (Masika et al. 1997; Moyo and Masika 2009). Dipping of cattle as a tick control method is not effective against ticks in sheltered locations such as between the toes, in the ears, or under the tail (Nicholson et al. 2019). Ticks that are missed, survive to reproduce rapidly and re-establish the pest population. Consequently, in this study farmers reportedly found actively growing ticks on cattle after dipping, and had to supplement the government provided dipping services with chemical acaricide sprays, pour-ons and injections with ivermectin. Nevertheless, the application of acaricides in form of sprays is also regarded as one of the practices that promotes acaricide resistance (Spickett and Firaz 1992). The acaricide spray technique especially when delivered using sub-standard equipment (locally fabricated sprayers), leads to uneven acaricide exposure (Jonsson et al. 2000). This subsequently, leads to an increase in the probability of ticks being exposed to declining acaricide concentrations, thus favouring the selection for resistance. Additionally, the practice of supplementation of the government-provided dipping services entails the adoption of a personal convenient tick treatment strategy, which may involve the use of higher treatment frequencies and doses of chemicals, resulting in the development tick-acaricide control resistance (Kumar et al. 2020).

This study found that synthetic pyrethroids (SP) were the most widely used acaricides in the control of ticks on cattle on the communal areas. This may be attributed to aggressive marketing and broad spectrum of vectors targeted by the chemical (Kumar et al. 2020). Unfortunately, the continuous use and inadequate application of SP over a long period (> 3 years) in communal areas of the ORTDM, could promote the selection of acaricide resistant ticks, thereby increasing the rate of resistance development (de Oliveira Souza Higa et al. 2015), and tick-acaricide control failure. Amitraz (Triatix®), a popular formamidine acaricide of Southern Africa (Rodriguez-Vivas et al. 2018) is no longer in use at dipping tanks on communal farms, probably due to reported tick-acaricide control failure (Strydom and Peter 1999; Mekonnen et al. 2002; Baron et al. 2015) or as a means of implementing acaricide rotation by the provincial government (Vudriko et al. 2018). This study noted an acaricide treatment frequency of 24–48 times/year during summer periods, whereas, dipping was highly inconsistent during winter, ranging from 0–24 times/year. Comparatively, at another communal farming community located at Wartburg of the ECP, dipping was done 24 and 12 times/year during summer and winter periods respectively (Nowers et al. 2013). Meanwhile, on commercial farms of the ECP, most farmers treated their cattle 21–25 times/year while a few treated their animals more than 41 times/year (Spickett and Fivaz 1992). The use of higher acaricide treatment frequencies as noted in the current study, is a likely indicator of tick control challenges (Spickett and Fivaz 1992). This practice increases the selection pressure on ticks over time, leading to the development of acaricide resistance (Rodriguez-Vivas et al. 2018). The probability of ticks developing resistance is higher when acaricides are used more than 5 times/year (Jonsson et al. 2000). Sutherst and Comins (1979) has recommended an acaricide treatment frequency of 16 times/year, especially during spring or early summer periods when a large proportion of tick populations are in the parasitic stage.

In this study, the alleged use of lower acaricide dosages for tick control at the dipping tanks was statistically associated with proportion of farms reporting tick-acaricide control failure. In a similar vein, Mekonnen et al. (2002) had indicated that the use of incorrect acaricide concentrations is one of the most important factors affecting the efficacy of acaricides at communal dipping tanks of the ECP. Although the mixing and replenishment of the dipping tanks was reportedly conducted by trained individuals, measurement errors could have led to inappropriate acaricide doses (Mekonnen et al. 2002). However, the alleged weak strength of the dip solution probably could have arisen from shortages in the government provided acaricides, mud accumulation and high water level inside the dipping tank after heavy rains. The exposure of ticks to lower acaricide doses favours the selection of resistant individuals (Kemp and Kunz 1994) and consequently to tick-acaricide control failure. Therefore, for optimal tick control on cattle that would lessen the effects of tick-acaricide control failure, farmers commanded for the application of higher acaricide doses. This finding corroborates that of Mekonnen et al. (2002), where farmers demanded for an increased in acaricide concentration in the dip tank during the peak tick season. However, when considering the selection pressure that may have already been imposed by the application of lower acaricide doses, any increase in the acaricide dosage would result in a rapid development of resistance (Kemp and Kunz 1994). Nevertheless, even if the high-dose strategy was to be considered, there would possibly be some limitations on their use including, uncertainties regarding the exact dose required to kill resistant ticks, restrictions related to the approved dosage for the product as well as animal and environmental safety issues (George et al. 2004).

It is interesting to note that the reported tick-acaricide control failure in the ORTDM could have been as a function of a whole set of acaricide application practices. For instance, approximately 50% of the communal farms exhibited combinations of at least four inappropriate control practices, including the alleged weak strength of the dip solution, irregular tick control, high acaricide treatment frequency, and failure to treat all cattle in a herd. Other sub-optimal tick control practices that predispose to tick- acaricide failure included poor structural state of the dip tank and incorrect rotation of acaricide. In addition, the fact that farmers could not remember the exact duration of acaricides used previously, probably also has an implication on the correctness of acaricide rotation. This study also noted that communal farmers generally practiced mixed livestock farming with cattle and other livestock combinations, mostly including sheep, goats and horses. These animals are alternative hosts to cattle tick species (Horak et al. 2018). Therefore, they should be considered together with cattle with regard to the control and eradication of tick species. Furthermore, it would be of interest to explore the reasons why communal farmers treated ticks on cattle at irregular intervals. We suggest that the encountered tick-acaricide control failure probably might have discouraged some farmers from participating in dipping programs. Nevertheless, irregular dipping of animals could have been due to shortages in acaricide solution or lack of water to replenish the dip as a result of drought (Eisler et al. 2003), farmers making use of other control options (Moyo and Masika 2009), elderly cattle owners’ inability to walk long distances for cattle treatment (Sungirai et al. 2016) and, the lack of repairs or structural maintenance of dip tanks (Eisler et al. 2003).

A limitation to this study include the purposive selection criterion that was used for the sampling of participatory farms. Such a criterion might have limited the ability to make generalisations on tick-acaricide control failure, as farms with smaller herd sizes (< 1000) were excluded. Additionally, a few pre-selected farms were excluded as they were not easily accessible. To limit this, questions were constantly evaluated and interpreted to be sure that they were understood correctly. The study was unable to verify the possible contribution of poor acaricide quality on the reported tick-acaricide control failure. Furthermore, the study did not undertake morphological or molecular identification of the tick species present on cattle. This is a crucial omission since different tick species may necessitate distinct strategic control measures.

## Conclusion

This survey confirms that prolonged and frequent exposure of ticks to inappropriate doses of the same acaricide chemical compound, leads to increased probability of tick-acaricide control failure, possibly due to emergence of tick acaricide resistance. Additionally, the likelihood of tick-acaricide control failure problem on communal areas of South Africa is expected, as approximately 50% of farms display combinations of at least four tick control malpractices. Nevertheless, the reportedly high rate (75%) of tick-acaricide control failure on communal farms should be investigated further to ascertain the manifestation of resistance development. Acaricide control failure management strategies should include educating field veterinarians and farmers on acaricide use and resistance in ticks to these chemicals. Farmers are urge to use acaricides strategically and judiciously, focusing on targeted applications in farms or areas where tick populations are concentrated. Furthermore, field veterinarians should be encouraged to identify and report tick-acaricide control failure promptly for early diagnosis, to promote the sustainability of the currently available acaricide chemical formulations.

### Electronic supplementary material

Below is the link to the electronic supplementary material.

Supplementary Material 1

## Data Availability

The datasets generated during and/or analysed during the current study are available from the corresponding author on reasonable request.
