# Supplementary material for: Risk factors contributing to tick-acaricide control failure in communal areas of the Oliver Tambo district eastern cape province, South Africa
Source: Exp Appl Acarol. 2024 Apr 24;93(1):17–33. doi: 10.1007/s10493-024-00910-x (PMC11182810; doi:10.1007/s10493-024-00910-x)
Supplement: Supplementary file 1 — Supplementary Material 1 [file 10493_2024_910_MOESM1_ESM.pdf]

Supplementary Material

Questionnaire on Risk factors contributing to tick-acaricide control failure in communal areas of the Oliver Tambo District Eastern Cape Province, South Africa

1. Date of interview……………….Farm/dip tank no…………GPS Coordinates ....

Local Municipality	Town	Village and address	
Name of Owner/ Sex: M / F	Contact no.	Occupation	Position on farm (farmer or committee member etc.)

2. Cattle rearing

Cattle breeds or cross breeds on your farm	Total number	Purpose

3. Other Livestock Keeping

Animal	Yes/No	Total number	Purpose
Sheep			
Goats			
Horses			
Others, mention			

Livestock Management system

4. What kind of vegetation is your livestock on?

Vegetation	Thornveld	Thornveld and forest	Grassland and forest	Grassland
Yes/no				

5. Do you have game/ wildlife animals on the farm? | Yes | No |

6. If Yes, do they come in contact with your cattle? | Yes | No |

7. Do your cattle interaction with those of your neighbors'? Yes No.

Daily	Weekly	monthly	Never

Ticks and tick-borne diseases.

8. Do your cattle have ticks on their bodies?

Yes	No

9. If **Yes,** what are the kinds of ticks? (place a tick where appropriate)

Bont ticks(banded legs)	blue ticks	brown ear ticks	red-legged ticks	Others

10. Do ticks cause problems in the cattle on your farm?

Yes	No.

11. Do you know the effects of ticks on cattle? (place a tick where appropriate)

Tick borne diseases	Anaemia	loss in weight	loss of appetite	ear damage	body damage	death

12. When do you experience greater number of ticks on cattle? (place a tick where appropriate)

summer period	winter period	whole year

Tick control

13. Do you control the ticks on cattle?

Yes	No

14. If Yes, what do you use to control ticks?

handpicking of ticks	use of chickens to remove ticks	chemicals (acaricides)	home-made remedies	pasture management	None	Others:

15. Which methods of application of chemicals do you use?

Dipping	spray races	hand spraying	spot treatment	pour-ons	injectable compounds	Others:

16. Which equipment is used to apply the chemicals to kill ticks on cattle?

dip tank	hand operated sprayer	back-pack sprayer	spray race	Other:

17. What do you use to restrain cattle during application of the chemical?

Cattle crush	holding yard in a kraal	ropes	a corner of the farm	Other:

18. If you use dip tanks, when was the last time you brought your cattle for dipping?

One week ago	Two weeks ago	One month ago	More than a month ago.

Sources of advice, and acaricide application practices

19. Who gives you advice on how to control ticks?

Vet drug shop teller	Veterinarian or Animal health technician (AHT)	Dipping committee member	Personal experience	Other:

20. From where do you obtain your chemicals?

Vet drug shop	Veterinarian or AHT	Dipping committee	Spar supermarket	CAHW	Others:

21. Who applies the chemical acaricides on cattle?

AHT/CAHW	Dipping committee member	Cattle owner	Other:

22. Who mixes the acaricides/chemicals before application?

AHT/CAHW	Dipping committee member	Cattle owner	Other:

23. The person who applies and mixes acaricides has a basic farmer training course

Yes	No

24. Water used to mix acaricides and fill diptanks comes from:

Nearby River	Rain	Dam	Tap	other:

25. How often do you control ticks on your cattle with chemical acaricides?

Summer/warm season	2-4 times a month	Monthly	Inconsistent/irregular
Winter/cold season	2-4 times a month	Monthly	Inconsistent/irregular

26. As a community, do you usually discuss about the best tick control methods for your communal area?

Yes	No

Acaricide treatment history and failure

27.

	Used currently	Used previously
Names of acaricide chemicals:		
For how long have you used/ been using the acaricide?		
Method of application (Tick them)	Plunge dip:<br>Pour on:<br>Spray:<br>Injectable:	Plunge dip:<br>Pour on:<br>Spray:<br>Injectable:
What made you to change to this particular chemical?(tick answers)	Vets/AHT/CAHW advice:	Vets /AHT/CAHW advice:
	Ticks not dying:	Ticks not dying:
	Shortage/lack of water	Shortage/lack of water:
	Other:	Other:

28. Do you usually notice any significant reduction/change in the number of active ticks on cattle after regular application of acaricides?

Yes	No

29. What do you do think should be done when acaricides fail to kill all ticks on cattle?

Increase the concentration	mix two different acaricides	Spray with another acaricide	Use plant extracts	Other; Specify

30. As a community, do you discuss strategies to deal with tick-acaricde failure?

Yes	No

31. Whenever acaricides fail, who do you consult for advice?

Vet shop	fellow farmer	AHT	Personal experience	Herdsman	other.

32. What do you think are the causes of acaricide failure to kill all ticks on cattle?

poor state of dip tank	Government supplied acaricide is weak	Other reasons:

Interaction of animals in the neighborhood and quarantine of newly introduced animals

33. Are there cattle interactions between treated and untreated animals in the communal grazing areas?

Yes	No

34. Do all farmers in the communal grazing area bring all their cattle all the time to be treated as required?

Yes	No

35. Do you usually introduce new animals into the communal area?

Yes	No

36. Are newly-introduced cattle in the communal area treated before being place among the rest of your herd?

Yes	No

37. Do you think the problem of acaricide failure problem coincided with the arrival of new animals?

Yes	No

Thank you for your cooperation.
